# Development and Testing of the Curiosity in Classrooms Framework and Coding Protocol

**DOI:** 10.3389/fpsyg.2022.875161

**Published:** 2022-04-07

**Authors:** Jamie J. Jirout, Sharon Zumbrunn, Natalie S. Evans, Virginia E. Vitiello

**Affiliations:** ^1^School of Education and Human Development, University of Virginia, Charlottesville, VA, United States; ^2^School of Education, Virginia Commonwealth University, Richmond, VA, United States

**Keywords:** curiosity, education, observations, instruction, protocol development

## Abstract

Curiosity is widely acknowledged as a crucial aspect of children’s development and as an important part of the learning process, with prior research showing associations between curiosity and achievement. Despite this evidence, there is little research on the development of curiosity or on promoting curiosity in school settings, and measures of curiosity promotion in the classroom are absent from the published literature. This article introduces the Curiosity in Classrooms (CiC) Framework coding protocol, a tool for observing and coding instructional practices that support the promotion of curiosity. We describe the development of the framework and observation instrument and the results of a feasibility study using the protocol, which gives a descriptive overview of curiosity-promoting instruction in 35 elementary-level math lessons. Our discussion includes lessons learned from this work and suggestions for future research using the developed observation tool.

## Introduction

Curiosity needs food as much as any of us, and dies soon if denied it.

-Stella Benson (*I Pose*)

Curiosity is widely acknowledged as a crucial aspect of children’s development, and as an important part of the learning process (Jirout and Klahr, 2012). Evidence suggests associations between curiosity and achievement at school entry (Shah et al., 2018) and that curiosity supports academic performance, even when controlling for students’ effort and ability (von Stumm et al., 2011). Despite this evidence, most prior research on the development of curiosity or on promoting curiosity has been conducted in lab settings with individual children (e.g., Cook et al., 2011; Gweon et al., 2014; Shneidman et al., 2016; Danovitch et al., 2021 among others), rather than in school settings. In research that did look at promoting curiosity in an educational context, researchers test specific manipulations with researchers administering the lesson to promote curiosity in schools (e.g., Lamnina and Chase, 2019) or parents in a museum setting (e.g., Willard et al., 2019), or observed children’s exploration without studying instruction or promotion of curiosity (e.g., van Schijndel et al., 2018). To our knowledge, measures of curiosity promotion in the classroom are absent from the published literature. We extend this prior work by focusing on more general practices that can be used to promote student curiosity in classroom contexts, developing a protocol for measuring this promotion, and showing its feasibility of use for future research.

There is concern that curiosity declines with education (Coie, 1974; Engelhard and Monsaas, 1988; Engel, 2015), so it is important to identify and provide what Benson refers to as the “food” for curiosity in educational contexts. In this methodological piece, we introduce the Curiosity in Classrooms (CiC) Framework coding protocol, a tool for observing and coding instructional practices that support the promotion of curiosity (Jirout et al., 2018). We describe the development of the observation instrument and the results of a feasibility study using the protocol with the intent to make the instrument available to the research and evaluation community.

### Defining Curiosity

A challenge in studying curiosity in education begins with the initial challenge of defining and operationalizing the construct of curiosity. Curiosity can be described as multidimensional, with theories suggesting different “types” or dimensions of curiosity (Kidd and Hayden, 2015), but it is also multifaceted in that it can include affective, cognitive, motivational, physiological, and expressive processes (Jirout and Klahr, 2012). For example, curiosity has been described as recognition that there is something unknown that one wants to know, or that there is ambiguity or uncertainty to resolve (cognitive; Jirout and Klahr, 2012), excitement or anticipation of pleasure from learning something new, or uneasiness of not knowing something (affective; Litman et al., 2005; Litman, 2008), and the desire to seek information by exploring or asking questions (motivational; Ryan, 2012; Pekrun and Linnenbrink-Garcia, 2012), all of which can be important influences in educational contexts.

Here we operationalize curiosity as a desire to seek information when something is unknown, and especially a preference to explore and gather information under conditions of uncertainty (Loewenstein, 1994; Jirout and Klahr, 2012). This definition stems from research showing that uncertainty leads to greater levels of exploration, with less exploration when there is too little or too much uncertainty (Loewenstein, 1994; Litman et al., 2005), and individual differences in uncertainty preferences, or the levels that an individual considers to be too little or too much (Jirout and Klahr, 2012). This assumption considers both a state aspect of curiosity, in that curiosity is momentarily sparked in response to the presence of uncertainty, as well as a more stable aspect of curiosity in the difference in preferred uncertainty for exploration, which can lead to higher likelihood to have curiosity sparked across contexts more generally. Rather than trying to disentangle the debate about curiosity being a state or a trait, or how it should be considered in both ways in education research (e.g., Engel, 2015; Murayama et al., 2019), we suggest that more regular experience of curiosity as a state can also lead to developing more stable curiosity, discussed further below. If curiosity includes both the desire for information and exploration to gather that information, regular promotion of curiosity in classrooms would result in more frequent feelings of curiosity and information-seeking behavior. At a more stable level of curiosity (i.e., individual differences), promoting curiosity would lead to greater comfort with uncertainty over time, so that preferences for exploring would relate to greater levels of information gaps (Jirout and Klahr, 2012; Jirout et al., 2018).

### Curiosity, Learning, and Education

In general, curiosity is associated with motivation and behavior that is conducive for learning, such as engagement and persistence in facing obstacles and setting goals (Kashdan and Steger, 2007), in developing sustained interests, which, in turn, can promote self-regulation, information-seeking, and motivation (Renninger, 2000; Hidi and Renninger, 2006), and with social, emotional, and cognitive development across the lifespan more generally (Kashdan, 2006; Kashdan and Steger, 2007; Keller et al., 2012). Aligned with associations between curiosity and learning, teachers have a positive perception of student curiosity and view its role in learning as distinct from traits, such as creativity and imagination (Chak, 2007). Further, teachers consider curiosity to be a didactic tool in the classroom, supporting better relationships with peers during group activities, encouraging critical thinking, and fostering feelings of self-determination (Menning, 2019). Teachers rate more curious children higher in competence motivation, attention, and persistence (Jirout and Klahr, 2012), and children with higher levels of curiosity are generally perceived by teachers as more likely to explore, share their interests with peers, and express excitement (Spektor-Levy et al., 2013).

Despite this extensive valuing and benefit of curiosity for learning, and as researchers and educators lament, children’s curiosity seems to diminish as they progress through formal education, at least curiosity expressed in school (Engelhard and Monsaas, 1988; Engel, 2011; Post and Walma van der Molen, 2018). Levels of expressed curiosity in students are very low by 1st grade, and almost completely absent by 5th grade (Engel and Randall, 2009). While research explaining this pattern is lacking, one suggested explanation of low curiosity in schools is an inconsistency between the emphasis on performance in schools and student curiosity (Engel, 2015; Jirout et al., 2018). For example, traditional instructional assignments often focus on getting correct answers or doing things the “right” way, leaving little room for students to question, wonder, or try out new or different ways of doing things. If this is true, it might be that children are not losing curiosity, but rather simply are not curious while in school. Such an argument is consistent with studies comparing children’s curiosity within and outside of school (Tizard and Hughes, 1984; Post and Walma van der Molen, 2018). For example, Tizard et al. (1983) assessed preschool children’s questions at home and in school and found that children asked more than ten times as many questions at home, with hourly rates of “curiosity” questions at home vs. school as 2.3 vs. 20.6 and 0.3 vs. 12.3 for middle- and working-class samples, respectively.

Why might children ask fewer questions and be less curious in school? In a recent study that surveyed preschool and elementary-aged children about what they were curious about, children rarely responded with curiosities related to school spontaneously, and, when prompted about what they were curious about in school, responses were mostly unrelated to investigative learning (Post and Walma van der Molen, 2018). Results also showed inconsistency between being curious and children’s perceptions of expectations in school in some reported responses. In the report, the researchers reported some children as “quite surprised or even disturbed when we asked them to share their school-specific curiosities,” with example responses of: “No one is curious about what we learn in class. We just need to do whatever the teachers tell us to do,” “It does not matter whether I am curious, because we just need to learn whatever we are assigned to do,” and “Are you joking? There is nothing to be curious about, when doing boring math or reading” (Post and Walma van der Molen, 2018, p. 65). With such significance for lifelong learning and wellbeing, it is important to better understand how curiosity is manifested in formal educational contexts and how curiosity can be promoted through instructional practices.

### Promoting Curiosity in Education

To explore the ways in which educational contexts can promote curiosity, it is important to first identify what it looks like to be curious in classrooms, and what that means for what a change in curiosity might look like. Based on the operationalization described earlier, we suggest that being curious in a classroom means that students recognize and feel comfortable with uncertainty, which leads to them becoming curious and engaging in exploration to find the missing information. Importantly, for students to feel comfortable being curious in a classroom setting, we hypothesize that they must have the perception that their teacher welcomes and values curiosity, and believe that curiosity is important for learning and has a place in school (Post and Walma van der Molen, 2018).

Our operationalization of curiosity as information-seeking in response to knowledge gaps (Loewenstein, 1994; Jirout and Klahr, 2012) describes the cognitive processes involved in curiosity and provides direction for identifying potential methods of promoting curiosity in education, including both individual student characteristics and contextual factors. When specifically considering individual factors, being more curious means having a higher preference or threshold for uncertainty, that is, being more likely to explore and seek information in the presence of larger knowledge gaps. Prior research suggests that the relation between uncertainty and curiosity follows an inverted U shape curve, where there is an optimal level of uncertainty that will lead to curiosity and exploration (Loewenstein, 1994; Jirout and Klahr, 2012; Kidd et al., 2012). For example, several studies measured children’s curiosity using a game in which children chose what they wanted to explore, with the choice between options that gave more information or less information (i.e., introduced more uncertainty) about what they would find; choosing to explore the option that presented more uncertainty indicated higher curiosity (Jirout and Klahr, 2012; Gordon et al., 2015; van Schijndel et al., 2018). Children showed individual differences in the level of uncertainty that led to exploration, and these differences were associated with convergent measures, such as question asking behavior and teacher-rated learning behaviors (e.g., competence motivation and persistence, Jirout and Klahr, 2012). Translating to an educational context, this might look like a student choosing a project they know less about over one in which they already know much of the information, or a student reading beyond what is assigned because they want to know more even after they have read the information needed to do an assignment.

We suggest that continuously promoting curiosity during instruction in ways that are positively experienced can help children to develop a more stable comfort with uncertainty, thus positively influencing their more stable curiosity over time. This is similar to the concept of the Broaden and Build theory (Fredrickson, 1998), which suggests that experiencing positive affect for learning can lead to an “upward spiral” of broadened cognition that further supports future positive affect (Fredrickson and Joiner, 2002) and to the reward-learning framework of curiosity and interest (Murayama et al., 2019). Murayama and colleagues suggest that engaging in momentary information-seeking can support future information-seeking more generally by reinforcing the behavior through the “reward” of gaining the valued information (i.e., an intrinsically rewarding feedback loop), as well as by expanding one’s knowledge base, which can lead to new questions and new opportunities to explore (Murayama et al., 2019; Murayama, 2022). This differs from what we theorize to occur in that it focuses on changes based on reinforcement through rewards. Our conceptualization of curiosity focuses on changes in the amount of uncertainty learners prefer. In other words, the reward-learning framework describes how the reward feedback loop promotes increased engagement in information-seeking when there is uncertainty they want to resolve, while we focus on learners’ decisions about what to explore, with possible options often varying across levels of uncertainty. Rather than seeing increases in general frequency of information-seeking with increasing curiosity, we would expect to see increases in the amount of uncertainty learners want to explore—whether they choose things that are more or less uncertain, and, thus, result in more or less that can be learned.

In our earlier work, we developed a framework identifying the means by which teachers can create a classroom climate that promotes students’ curiosity through instruction and language, as well as ways curiosity might be suppressed: The Curiosity in Classrooms (CiC) framework (Jirout et al., 2018). The CiC framework suggests two ways by which instruction might promote curiosity: (1) helping students to recognize and become more comfortable with uncertainty (initiating or sparking curiosity), and (2) helping children learn to seek information to resolve curiosity (promoting curious behavior—that is, exploration and information-seeking). Importantly, the CiC framework illustrates how the instructional language teachers use to present content and learning activities might promote or suppress curiosity. This approach aligns with the process–product model of Brophy and Good (1984), which has been successful in prior research identifying effective contextual factors and teaching practices. For example, studies using this model have shown that classroom climate, and specifically teacher interactions, can create a nurturing and supportive environment for students to learn (Hamre and Pianta, 2005). Curiosity and learning beliefs are likely to be highly sensitive to specific language used, and, importantly, these factors are malleable. For example, the growing number of interventions related to the construct of mindset demonstrates its plasticity, and teacher language could be an important influence of developing mindset beliefs about learning (Yeager et al., 2013). For example, children who learned from a robot that modeled a growth mindset during instruction, compared to a control robot that did not model growth mindset, agreed more strongly with growth mindset statements (H. Park et al., 2017). Similarly, children who learned from a “curious” robot teacher—one that asked probing questions—scored higher on curiosity and exploration tasks than those whose robot teacher gave the same information without questions (Gordon et al., 2015). These studies show the effect of language can have on curiosity and learning attitudes.

The CiC framework of instructional practices to promote curiosity is presented with examples in Table 1, and a detailed explanation of prior theoretical and empirical literature supporting the different framework components can be found in our past work (see Jirout et al., 2018). However, the framework development was influential in describing the development of our observational tool and is not described elsewhere, so in this work, we begin by describing the approach we used to identify specific instructional practices that can promote curiosity. We then focus on the primary goal of this work: to develop a coding protocol for and test of the framework.

**Table 1:** Curiosity in classrooms framework: types and examples of curiosity-promoting instruction.

Curiosity promotion		
Categories	Practice	Example
C1: Promoting feelings of curiosity (recognition of and desire to explore uncertainty)	C1.1 Opportunities to think, question, and participate	“Take a few minutes to look at the image, and think about what you notice or wonder.”
	C1.2 Modeling positive reactions to uncertainty	“You know, sometimes I get confused, too.”
	C1.3 Prompting question generation	“Who can share questions we could ask to learn more about this?”
	C1.4 Reviewing known and unknown information and making connections	“We know that alligators are reptiles. What do we know about reptiles? What might that tell us about how alligators live?”
	C1.5 Encouraging alternative ideas	“Who did something different—can someone share another way we could try to solve this problem?”
C2: Promoting curious behaviors (exploration and questioning to resolve uncertainty)	C2.1 Opportunities to explore ideas, materials, and questions	“Now that you have the cubes, try and use them to explore different ways to show fractions.”
	C2.2 Scaffolding information-seeking	“I bet you can find that out—what could we search for on Google that might have some information?”
	C2.3 Positive verbal and non-verbal responses to students’ questions	“What an interesting question!”
**Curiosity Suppression**		
S1: Avoiding uncertainty and promoting discomfort with uncertainty		“I’m not sure why it looks different but we need to move on, so just pay attention to the picture in your book for what it should look like.”
S2: Actively discouraging information-seeking behaviors		“Your materials do not look like you are following the instructions; stop playing around and focus on the question you are supposed to answer.”

## Materials and Methods

### Design and Development Process

**Figure 1:**
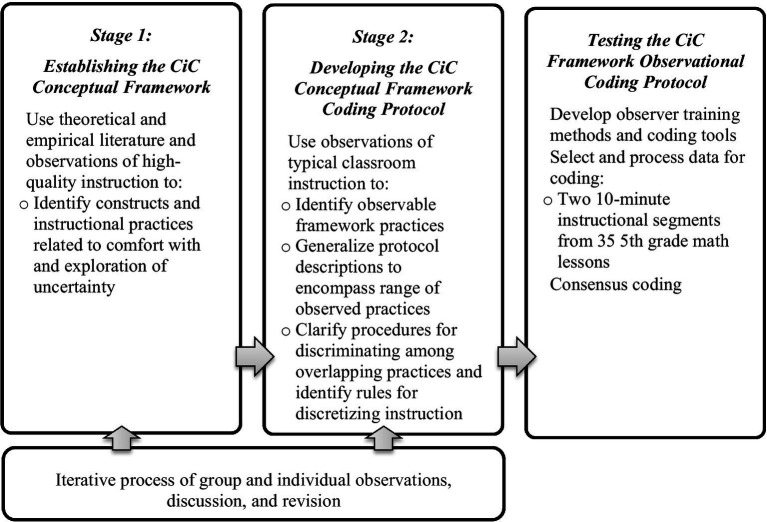
Overview of the CiC framework and protocol development process.

The CiC framework is a set of instructional practices identified to promote curiosity, while the CiC framework coding protocol is a guide describing how to identify practices observed during instruction with detailed specifications for what is and what is not included within each of the framework elements. The CiC framework and coding protocol development involved two stages with several steps within each to identify and measure instances in which curiosity could be promoted or suppressed by teachers (see Figure 1).

#### Stage 1. Establishing the CiC Conceptual Framework

The development of the CiC framework and identification of instructional practices that either promote or suppress curiosity began with an empirical literature review (see Jirout et al., 2018) and observations of video-recordings of classroom instruction from prior unrelated studies and from online resources, such as teachingchannel.org. A multi-disciplinary team of researchers with expertise in studying motivation, curiosity, effective K-12 pedagogy, and/or developmental psychology and in developing observational tools met across multiple sessions to watch and discuss instruction with the goal of observing behaviors relating to the theoretically identified categories of curiosity promotion and suppression based on the Information-Gap Theory (Loewenstein, 1994). From these observations, many behaviors with the potential to positively or negatively influence student curiosity were identified. These were then grouped by theme, resulting in the promotion and suppression categories (see Table 1).

In describing what curiosity promotion might look like in classrooms, we focused on specific ways that students might become more comfortable with uncertainty, practice recognizing uncertainty, and learn to and practice resolving uncertainty, and the corresponding teacher supportive behaviors for each of these categories. As such, we divided curiosity promotion into two categories: promoting feelings of curiosity (C1) and promoting curious behaviors (C2), aligned with eight practice types across the two categories. We also identified two categories of curiosity suppression: promoting discomfort with or avoiding uncertainty, and actively discouraging information-seeking behaviors, each aligned with one practice type. In the following sections, we describe categories and associated practices in more detail.

##### C1. Promoting Feelings of Curiosity

The category of *promoting feelings of curiosity* addresses teacher actions and utterances that create an environment supportive of students’ curiosity. This is done by promoting a climate where students feel safe taking risks, making mistakes, failing, and not knowing or not being sure (Dess and Picken, 2012; Jirout et al., 2018). Teachers’ behavior and language can promote this type of classroom climate by providing opportunity and encouragement for these behaviors, as well as modeling the behaviors in themselves (Zimmerman and Pike, 1972; Sullivan, 2011). This category looks specifically at how the teacher prompts students to be curious and guides children on how to be curious. Actions and utterances within this category promote curiosity (and children’s learning to be curious) rather than promoting and developing skills involved in resolving curiosity. Specifically, five instructional practices were identified as methods of promoting students’ ability to recognize uncertainty or to develop greater comfort with it.

First, to promote feelings of curiosity, teachers can provide students with opportunities to think, question, participate, and respond, such as having students take a moment to think about something and share it with a peer before having a student respond to the whole class, allowing broader participation and engagement (Rowe, 1972). For example, asking questions that guide children’s thinking rather than giving them information is found to support exploration and broader learning (Bonawitz et al., 2011; Yu et al., 2018; Jean et al., 2019). Providing opportunities for students to become curious can promote their engagement in learning, as suggested in a study where students were more likely to explore hints about information they were curious to know then to just get the answer (Metcalfe et al., 2021). Related to this, a second approach teachers can take is to elicit multiple responses from several students, allowing them to hear each other’s perspectives and ideas and showing that there are different ways of thinking about things (Duschl and Osborne, 2002). For example, students instructed to seek differing ideas from other students in a science class learned more than students who were asked to seek ideas from classmates that matched their own (Matuk and Linn, 2015). Common activities, such as Think-Pair-Share, can support these opportunities for children to become curious, active participants in their learning (Rowe, 1986; McTighe and Lyman, 1988; Ciardiello, 1998), and simply waiting a few seconds longer after asking questions can increase the number, types, and quality of student responses (Rowe, 1972). Third, teachers can model their own comfort with uncertainty or mistakes, showing a sense of wonder about something they do not know, or pointing out the benefit of learning something new when exploring what led to a mistake (Engel, 2015), which can also broaden their own thinking about the material (Zimmerman and Pike, 1972; McDonald, 1992). When teachers focus more on knowing how to find information and are comfortable acknowledging the limits to their knowledge, they are more open to student interests, and students have deeper and more meaningful questions and discussions (Cunnigham, 1997). Benefits of a “motivation culture” in classrooms are found for both motivation and achievement, and stronger perceptions of mistakes as useful for learning are associated with higher motivation (Käfer et al., 2019). Fourth, teachers can help to support the metacognitive process of considering what is known related to a question, and what is unknown or what information is needed, helping children to explicitly recognize knowledge gaps (King, 1994; Rosenshine et al., 1996). Fifth, students can be explicitly prompted to generate questions, different from simply asking “are there any questions?” but rather giving students the task of thinking of what questions they could ask to learn more (e.g., “what questions could we ask to learn more about this?”). Having students’ own questions included in the learning process can support a sense of autonomy for learning, which is a powerful motivator for learning (Grolnick and Ryan, 1987). Together, these methods can all help to support a learning culture where mistakes are not seen as something to try and avoid (Martin and Marsh, 2003; Martin, 2011), and instead puts the focus on the learning process whereby inquiry is valued and appreciated.

##### C2. Promoting Curious Behaviors

The second category of curiosity promotion, *promoting curious behaviors*, includes language and behavior intended to provide the opportunities, instructions, and reinforcement of manifestations of curiosity, (i.e., curious behaviors including information-seeking, such as exploration and asking questions). This category includes teacher actions and utterances encouraging students to act on their curiosity, as a result of either the classroom settings/provocations, teacher verbal and non-verbal responses/prompts, and/or explicit communications about how to be curious. A key distinction between the C1 and C2 categories is that C1 refers to teacher language and behaviors that might influence children’s *becoming* curious by recognizing and preferring uncertainty, while C2 refers to teacher language and behaviors that might develop behaviors to resolve uncertainty, supporting students’ information-seeking strategies and skills. To promote curious behavior, learning experiences can include opportunities to explore materials, ideas, and questions without concrete steps to follow, such as in a “tell and practice” approach, which research shows can “overemphasize efficiency at the expense of discovering new ways of seeing and doing” (Schwartz et al., 2012). This allows students to practice identifying the specific problem they are trying to solve and ways of solving it, with focus more on the process of exploring and learning over the end-goal of finding the answer (Dean and Kuhn, 2007; Sullivan, 2011). A second method of promoting curious behavior is to provide scaffolding to guide students’ information-seeking, helping to make knowledge gaps less intimidating by breaking down steps to find information or even to help identify information needed and ideas for ways of getting it (Turner et al., 1998; van de Pol et al., 2010). Support for focusing on this learning process can be important for developing openness to uncertainty, as a strong focus on outcomes over the process is negatively associated with children’s developing motivational framework (Park et al., 2016), while focusing on effort positively impacts children’s motivational frameworks (Gunderson et al., 2013). Finally, teachers can simply provide positive reinforcement to students’ curiosity and question asking, which can help create a classroom climate where curiosity is valued (Spargo and Enderstein, 1997; Pianta et al., 2008). Research shows that process praise (as opposed to praise for performance) is associated with children focusing on learning for the sake of learning over academic performance (Gunderson et al., 2018).

##### S1-2. Curiosity Suppression

While the absence of curiosity promotion may not have a negative impact on curiosity, we identified two instructional practices as potentially suppressive of students’ curiosity. The first category of curiosity suppression includes instances whereby teachers model avoidance or discomfort with uncertainty or mistakes or make comments that suggest to students that mistakes should be avoided. Teachers’ responses to student mistakes show how these responses can influence students’ perceptions about errors (Käfer et al., 2019). For example, Tulis (2013) identified and measured teacher responses to students’ mistakes as maladaptive or adaptive. The former, which included responses like criticism, negative affect (e.g., grimacing), or humiliation, made up about one third of teacher responses to mistakes, and there were rarely instances of reinforcing ideas around mistakes as learning opportunities. Not surprisingly, students tended to show negative affect when responses to mistakes, included humiliation, ridicule, or criticism (Tulis, 2013). On the other hand, students’ perceptions of classroom climate as accepting of mistakes are associated with higher motivation (Käfer et al., 2019).

The second category of curiosity suppression includes teachers’ discouragement of information-seeking behavior, such as negatively responding when students might try to do something in a different way than instructed or asking questions that went outside of the scope of the teachers’ learning goals (Carlsen, 1991; Engel, 2015). Both students and teachers described barriers to question asking at school including questions being perceived as unwelcome interruptions or getting negative responses to questioning (Dogan and Yucel-Toy, 2021). Importantly, this category does not include teacher redirection to focus students’ attention on instruction, as we see this practice as general classroom management to keep students from going off-task rather than curiosity suppression.

#### Stage 2. Developing the CiC Conceptual Framework Coding Protocol

##### Developing and Refining Initial Items

In developing items for coding observations based on the CiC framework, we used a similar iterative observation procedure with our team observing a standardized set of video-recorded classroom instruction from an unrelated research study. The team had weekly discussions while watching videos together about what types of language and behavior aligned with which framework practices, and how to disambiguate practices that were overlapping. For example, when a teacher asks students to think of alternative strategies or examples after an initial response is given, this could be considered an additional opportunity to think and participate, but is better fit as encouraging alternative ideas because it is more specific. We decided on a rule to code each instance as only one category, but to use the most specific category that fit what was observed. The specific examples of practices were recorded, along with detailed descriptions of the practices that were updated with discussions to create the CiC framework coding protocol.

##### Revising the Protocol to Be Comprehensive and Specific

After reaching agreement in the group observation coding sessions, the development team coded an additional set of five videos independently, and then met to discuss what was coding and further refine the protocol to include what was and what was not coded under different categories, and information about common questions and how coding decisions were made, all of which was included in the protocol document revisions. The independent coding followed by group discussions was an iterative process, continuing with about five additional videos in each session until the group discussions revealed agreement across coders. This resulted in a protocol that includes background literature and explanation of the purpose of the protocol, and an explanation of each framework component with detailed descriptions of the component, examples of what is coded as that component, and examples of what is not coded under that component. Additional notes were added based on the iterative discussions that revealed nuances to the coding protocol and helpful considerations for achieving reliable coding.

When coding, language excerpts were only included in one curiosity category. As discussed above, it was necessary in some cases to choose the *best* fit among the available categories, identified by alignment to and specificity of the category, with preference for higher specificity. In other instances, the same instructional activity might include more than one category if there are separate actions or components, for example, a teacher asks students to generate questions with a partner, and then has them share the questions as a whole group and write examples on the board [C1.3: prompting student questions; C1.1: opportunities to participate (pairs); C1.1: opportunities to participate (whole group)]. Likewise, instruction could be coded under more than one category if it contains multiple distinct components. For example, a teacher might say “Great question! Can anyone else think of more questions about this?” (this would be broken into two segments, coded as C2.3: positive response to a question and C1.3: prompting student questions). Note that not everything that a teacher says or does was coded; only instances that fit within the curiosity and motivation categories were coded. The full coding protocol is publicly available on databrary.org.^1^

##### Observer Training

Once agreement was reached by the development team and the protocol was finalized, a new team of data coders were trained to use the protocol to test the feasibility of using it for future research. Training coders to use the CIC was a multistep process. The first step involved having them read the protocol and test themselves on the specific categories using provided written practice activities and keys. Once coders were confident in understanding the individual categories and knew the differences across categories, they watched specific examples of classroom instruction using the protocol overview to practice recognizing instances of different categories, reviewing the protocol as needed. Coders then tested themselves using two training videos, involving coding videos using the protocol and then checking their codes against provided keys, which provide explanation for why different instructional components are coded (or not) as specific different categories. If they had mistakes or did not understand codes on the key, they completed an additional training video after more training.

##### Coding Methods and Efficiency

This initial work used consensus coding for reliability, in which multiple trained coders code the same videos independently, and then meet to ensure consensus and discuss any discrepant codes until 100% agreement was reached. We chose this method because the sample in the feasibility test was planned to be small, and we wanted to have the opportunity to evaluate and revise the protocol if needed. However, we designed the protocol with plans for using another more efficient method in future work, with individual coders trained to reliable coding standards. For example, the coders might code three test videos and have these scored by the protocol developers against a key, achieving a minimum of 85% accuracy of coding. Accuracy would be determined by the match between frequency across categories with the key. Then, reliability would be assessed across coders by having a subset of videos coded by multiple coders so that the consistency across coders could be assessed. Consensus coding is labor-intensive, so ideally reliability coding will be used in future work. Table 1 describes the coding categories used with example instructional language for each coding category.

### Testing the CiC Framework

#### Video Data Sources

Our data source for this initial test of the CiC framework used a convenience sample, the Measures of Effective Teaching (MET) database (Bill and Melinda Gates Foundation, 2009-2011), a corpus of classroom videos developed for researchers to study how instruction can be most effective for student outcomes. Because our focus of the coding was on instructional language, it was critical that videos have adequate audio data to clearly hear what is being said. Many constructs that were coded required some understanding of the general activity and/or context of the instruction and lesson, so having a view of materials, teacher gestures, projected material or whiteboard, etc. was beneficial to having a coherent understanding of the instruction for coding. For these reasons, we included videos rated as high-quality across all A/V quality ratings by MET research staff for teachers who consented for secondary data analyses (if teachers had multiple videos fitting all criteria, we used the earliest one). Finally, we only included videos of instruction collected in year 2 of the MET study, so that we could use student survey items that were only collected in year 2. These filters resulted in 35 videos.

#### Goals for the Feasibility Testing and What Was Coded

Our goal in coding with the CiC protocol was to estimate the frequency and range of experiences that students have related to promoting and suppressing curiosity. We aimed to develop a protocol that could be used in a relatively short amount of time, but that would be representative of what students in a classroom *typically* experience. In the future, we expect this coding method to be useful for real-time coding in classrooms. For the current work, we coded 10-min time segments from each lesson, with many lessons including two segments that were averaged, including one from the beginning and one from the middle of the lesson.

#### Selecting and Preparing Observations for Coding

Because our initial goal was to test the framework and whether it could be used to reliably code for curiosity promotion, we focused on a single domain to limit content-specific variability. Most research has examined curiosity as a domain-general construct, but recent work proposes that curiosity should be examined in domain-specific contexts (Peterson and Cohen, 2019). Understanding domain-specific curiosity may better inform how curiosity leads to learning in domains, such as math or science. In science lessons, for instance, recognizing and responding to uncertainty can drive science learning (Manz and Suárez, 2018; Lamnina and Chase, 2019). In math, highlighting knowledge gaps and providing opportunities to explore may be particularly important (Peterson and Cohen, 2019; Rumack and Huinker, 2019). As the current framework examines several specific curiosity-promoting practices, it can potentially be used to examine both domain-general and -specific curiosity.

We chose math as the first domain to investigate and focused on upper-elementary grades for several reasons. First, interest in and attitudes about academic subjects and ability, including stereotype ideas about gender differences, develop very early. By early elementary grades, children already show stereotypical gender beliefs of a stronger association between boys and math than girls and math, with girls showing a weaker self-identification with math than boys (Cvencek et al., 2011). Thus, finding ways of improving math instruction and motivation in learning could provide beneficial implications for addressing the problem of encouraging girls to pursue math and math-intensive careers. Additionally, math interest declines for all students between late elementary and middle school, though girls begin at much lower interest levels than boys in 4-5th grade (62% of girls and 95% of boys giving a positive interest response; (LeGrand, 2013). Teachers’ enthusiasm is associated with math interest even during the periods of math interest decline observed in adolescence, suggesting the important role of teacher practices (Frenzel et al., 2010). We were therefore interested in the role instruction might play in promoting children’s curiosity at the critical point immediately preceding the transition from elementary to middle school (4–5th grade), when interests have been observed to decline. To control for the difference in opportunity to promote curiosity and motivation based on age and topic, we selected videos within a single grade level that were focused on a specific topic: adding and subtracting fractions. Because understanding teacher language was necessary for our coding protocol, we selected videos that were identified by the MET project as high-quality across all A/V quality ratings by MET research staff for teachers who consent for secondary data analysis (if teachers had multiple videos fitting all criteria, we used the earliest one), and then a research assistant screened videos to ensure adequate quality to hear and understand the teacher for two, 10-min segments.

Segments were selected to begin at points in which a teacher was beginning instruction on content—we did not begin a segment while the teacher was asking students to take out their homework or textbook, or if the teacher was reading the information about the video recorders, etc. We also ensured that the 10-min segments did not include any type of non-typical activity, for example, a test during which there would not be an expectation of instruction, and/or something that was not expected to occur more than once a week. These activities were labeled as uncodable time, and if they occurred, the 10-min segment was selected to begin after the uncodable time ended. A single coder prescreened all videos and identified the codable segments by time points to begin coding, which were then used by all coders. Because videos were selected based on having high ratings of both audio and video coding, most teacher language was audible and codeable.

To begin exploring the validity of the coding protocol, we looked at questions students asked in the coded segments to test if they were associated with frequency of curiosity-promoting practices. Student questions were transcribed from a subset of videos as possible (e.g., audio quality was sufficient for student voices), resulting in 57 total student questions asked across 45 coded segments (*N* = 23 lessons; one lesson only had one 10-min segment with sufficient audio).

## Feasibility Test Results: Curiosity Promotion

Two 10-min segments were successfully coded from each of 35 5th-grade math lessons on adding and subtracting fractions using the video screening method described above. In general, and as many would suspect, we observed extremely few instances of curiosity-promoting language. Table 2 presents the total number of times that each curiosity-promoting type was observed across all videos, the mean frequency of observed instances within a lesson (two coded segments), the range of frequency observed across lessons, and the percentage of teachers who had at least one occurrence observed. While most teachers were observed using one or more instances of promoting recognition of or comfort with uncertainty (83%), the frequency of engaging in this across the two coded segments of a lesson was only 2.03 instances (*SD* = 2.1). Instruction that included opportunities to be curious, such as opportunities to “figure out” or positive reinforcement of curious behavior was only observed in 23% of teachers, with a mean of only 0.3 (*SD* = 0.63) instances total across the lessons observed. Most striking, we did not observe a single instance of teachers prompting students to generate questions across all videos coded. Note that a teacher simply checking understanding by asking, “any questions?” was not coded; rather this code was for explicitly prompting students to generate questions (e.g., “what questions do you have?” or “can you think of any questions we could ask?”). These low numbers indicate that promoting curiosity promotion was infrequent, but curiosity suppression was even rarer, with only 9% of teachers having any instances, and occurring 0.23 (*SD* = 0.91) times per lesson observed, meaning we only saw 7 instances across three of the 35 observations.

**Table 2:** Observations of curiosity-promoting instruction across all lessons coded (two 10-min segments from each of 35 lessons).

Coding Category:	Total (all) observation	Mean frequency per segment	Range of frequencies	% of teachers (any coded)
C1.1 Provide opportunities to think, question, participate	45	1.29 (SD = 1.89)	0–10	60%
C1.2 Modeling own comfort with uncertainty	3	0.09 (SD = 0.28)	0–1	9%
C1.3 Prompting question Generation	0	0	0	0%
C1.4 Reflecting on student prior knowledge and uncertainty	8	0.23 (SD = 0.49)	0–2	20%
C1.5 Encouraging alternative Ideas	15	0.43 (SD = 0.92)	0–5	31%
**C1: Average of promoting comfort with uncertainty**		2.03 (SD = 2.13)	0–11	
C2.1 Provide opportunities to explore and “figure out”	3	0.09 (SD = 0.37)	0–2	6%
C2.2 Scaffolding and guidance in resolving uncertainty	1	0.03 (SD = 0.17)	0–1	3%
C2.3 Positive responses to questions asked	7	0.20 (SD = 0.47)	0–2	17%
**C2: Average of supporting exploration and questioning**		0.31 (SD = 0.63)	0–2	
S1: Promoting discomfort with uncertainty	1	0.03 (SD = 0.17)	0–1	3%
S2: Negative responses to curiosity and information-seeking	7	0.20 (SD = 0.87)	0–5	9%

As an initial exploration of effects of curiosity promotion on students, we tallied questions asked by students during the coded segments. Surprisingly, we did not observe a relation between curiosity-promoting language and student questions in class. When we explored further, we found that when students heard no curiosity-promoting language, they asked an average of 1.4 (*SE* = 0.12) questions; when they heard only one or two instances of curiosity-promoting language, they only asked 0.4 questions (*SE* = 0.11); however, when they heard more than two instances of curiosity-promoting language, they asked an average of 2.1 (*SE* = 0.14) questions. It is important to note that this is at the classroom level—two questions means two questions asked *by the entire class*, in the whole 10-min segment. In fact, of the 45 segments for which we were able to transcribe student questions, more than half (24 total) had no student questions asked. Analyzed at the class-level, these differences are not significant. However, we only counted the frequency of questions and did not code questions by type, and coders reported that many questions were simply asking permission or clarification, so may not have been indicative of student curiosity.

## Discussion

Prior research shows a need to promote students’ curiosity, and for explicit attention to practices that create a curiosity-supportive classroom climate, such as concrete support for and encouragement of curiosity in students (Post and Walma van der Molen, 2018). Classroom climate is impacted by students’ observations of what teachers care about, which can be portrayed both indirectly and directly, such as through instruction (Jirout et al., 2018). As Katz notes, “...Even very young children are most likely making inferences about what adults care about based on multiple observations of the adults’ actual behavior in context” (Katz, 1998, p. 38). This paper described our process of developing a framework and observational tool to study how specific instructional practices can promote students’ curiosity by supporting their comfort with and recognition of things they do not know, which can help to promote their becoming curious, and by developing their information-seeking skills which can promote their curious behavior and learning. Although many studies have focused on individual aspects of instruction that might independently support curiosity, much of this research occurred in lab-based research and involved manipulating instruction. The CiC framework coding protocol will allow for understanding what is happening in actual classrooms and the study of how instructional practices impact student curiosity in these classrooms.

This framework and protocol are not intended to reveal what a “good” teacher or “good” teaching looks like; rather, our goal was to understand what instructional practices could occur that support developing student curiosity specifically. We tested the feasibility of using the developed tool for observing for curiosity promotion and described how frequently instructional practices related to promotion and suppression of curiosity occur. In addition to the protocol itself, we described this process of developing it and the results of the feasibility test. We discuss each of these components below, followed by suggestions for future research to validate and use the developed tool.

### CiC Design and Development Process

There are not currently well-developed methods of assessing what teachers do (or do not do) to support student curiosity, which limits the ability of research to support educational promotion of curiosity and research on the value of doing this. The work described here takes the initial step toward understanding the influence on instruction on student curiosity by developing a method for measuring instructional practices that might promote student curiosity to assess what works and whether it matters for motivation and learning. This work began with a process of integrating prior theory and research to align with our operationalization of curiosity to understand what changes in curiosity in a classroom context would look like, which included identifying the two types of support we include: support for becoming curious (i.e., identifying things one does not know and wants to find out) and support for being curious (i.e., seeking information). We then used an iterative process of observations, discussions, and alignment to prior work to identify the items in the CiC framework using instruction identified as high-quality, followed by a similar process using observations more representative of typical educational contexts to explain in detail what the framework practices could look like in observations of classroom instruction and methods for training observers and conducting these observations.

The process of developing this framework and protocol was difficult, and we hope that in addition to the contribution of the CiC itself, that the description of this process will also be helpful for researchers. Specifically, we would summarize our experience in three lessons learned for future efforts in similar work. First, operationalizing the construct of interest and being specific in what we wanted to observe was important. Although curiosity seems like a simple and common construct, it is poorly understood with definitional and measurement challenges (Jirout and Klahr, 2012; Kidd and Hayden, 2015). Despite these challenges, we used a specific operationalization of curiosity linked to learning behaviors to consider how student curiosity can be influenced by instructional practices. This operationalization focused our efforts around identifying practices related to developing comfort for and recognition of uncertainty and information-seeking, which helped to identify and categorize themes among the instruction practices that we observed in developing the framework. A second lesson was that developing this protocol was a much more iterative process than we originally anticipated. Instruction varies greatly from teacher to teacher and classroom to classroom, and it took many revisions of our framework descriptions and protocol instructions to become a generalization of instructional practices that aligned to the framework while still reflecting the many different types of instruction that might fit each practice. Our third lesson was related to the second: this was not a quick development process that could be assumed to be easily completed at the beginning of a larger project. This process was extensive, lasting about 18 months, because of the need for countless discussions, iterations, and revisions. Had we been pressured to complete the process of developing the protocol to conduct research using it, we may not have been able to spend the needed time and effort in its development.

### Results of the Feasibility Test

We acknowledge that the work here is presented as a very initial step in understanding the role of curiosity in natural settings, in that our current work aimed only to test the protocol and describe what types and frequency of curiosity promotion was observed in a single domain and grade level. Future work to validate the tool is necessary. That said, observations using the developed protocol showed low levels of curiosity-promoting instruction in a small feasibility test, despite prior research showing broad agreement that curiosity is valued and important in learning contexts. The practices observed were focused on ways that teachers promoted comfort with and desire to explore uncertainty. Children need opportunities to become curious and practice being curious, and this can happen by supporting and promoting students’ comfort with uncertainty (Jirout et al., 2018). If students are expected to listen and learn information without pauses to think about the information and ideas, they would not have the time needed for reflecting beyond what it is they heard to consider what it is they do not know but could be curious about, what Glăveanu (2022) refers to as “uncertain knowing.” Further, supporting a mindful approach to thinking about uncertainty can help to open children’s thinking and reduce the focus on worrying about judgment (Henriksen et al., 2022). The observational protocol described here, based on the CiC framework, will allow future research to explore what kinds of curiosity-promoting instructional practices are happening, how frequently, and whether those practices are linked to student outcomes. Further, the framework provides specific instructional practices that can be individually tested and explored in controlled studies for their efficacy in influencing students’ curiosity to develop concrete practical implications for what educators can do to support students’ curiosity, such as using “structured uncertainty” to support curiosity (Beghetto, 2020), where scaffolding and support is built in for students to practice thinking in new or different ways about a problem.

### Importance of and Need for Research on Curiosity Promotion

The findings of Post and colleagues (2018) that students do not see being curious as consistent with learning in school is troubling, but it offers a clear need for future research to understand how we can change this perception. Promoting students’ curiosity to learn in educational contexts could make learning more enjoyable and support motivation (Midgley et al., 2001), which could support future learning (Jirout et al., in review), and it also could positively influence learning behaviors, such as question asking and class participation (D. Park et al., 2017; Jirout and Klahr, 2020). This might be especially important and effective for domains like science (Jirout, 2020), where children’s ability to ask and think about questions is seen as fundamental, with the inclusion of question asking as the first of eight scientific and engineering practices that span across all grade levels and content areas in the National Research Council’s National Science Education Standards (NRC, 2012). Although the current test only looked at math instruction, we were still surprised not to have observed a single instance of teachers prompting students to generate questions, which could be an important way to help them recognize things they might be curious to know. In exploring their own curiosity, students might also develop more sustained interests in topics (Hidi and Renninger, 2006), which can support learning more complex material and contribute to learning beyond curiosity (Hidi and Renninger, 2019). In student self-reports of their interest across domains, science has the lowest proportion of being considered most interesting compared to math and reading, and this is significantly lower for females than males and for students eligible for free and reduced school lunch compared to higher-income peers (U.S. Department of Education, 2015). Importantly, these students’ ratings of interest in science are significantly associated with their performance (U.S. Department of Education, 2015). Developing curiosity could help to promote learning across domains and could especially support retaining and motivating students in pursuing science careers.

### Limitations and Future Directions

Although we observed low levels of these practices, we looked only within a narrow scope, assessing a small sample of lessons within the single domain of math, so we caution readers in drawing too much meaning from the data presented, which was intended more as a test of feasibility of using the protocol than a description of what is currently happening in classrooms. Future work should look more broadly at different educational levels and across different academic subjects and types of instruction (e.g., whole class and small group). Just as there is a need to explore whether students’ curiosity is domain-general (e.g., Loewenstein, 1994; Markey and Loewenstien, 2014), domain-specific (Peterson and Cohen, 2019), or, as we expect, reflects evidence of both domain-general and domain-specific curiosity (Murayama et al., 2019), it is possible that curiosity promotion might look different across domains or even across education levels.

We also only provide descriptive information about what we observed. While we attempted to look at associations with student questioning, there were too few questions asked by students across the observed lessons. Future research should explore what outcomes are predicted by curiosity-promoting instructional practices, especially looking at changes in students’ curiosity and related constructs, which was not possible using the convenience sample included here. Additionally, there are likely other important factors to consider in this future work. Though many metacognitive and social–emotional factors are important for children’s successful learning, this paper focused on curiosity as one such factor in that it can both move one to act and direct behavior toward finding specific information (Wentworth and Witryol, 2003). Curiosity can help to develop sustained interests (Hidi and Renninger, 2020), and, in turn, promote self-regulation, information-seeking, and motivation (Renninger, 2000; Hidi and Renninger, 2006). There are likely many other additional factors that are also important for curiosity and learning, such as mindset, achievement goal orientation, self-efficacy, academic courage, and intellectual humility. Understanding how curiosity relates to and differs from these different constructs is another important area for future work. In addition, there are likely important aspects of the social context of classrooms and peer interactions that also can influence curiosity (Käfer et al., 2019), as well as classroom resources and instructional design factors that may differ, such as use of and familiarity with technology. We hope that this work advancing methods of studying curiosity promotion in instruction will be useful in future research to explore these factors and the associations among them to understand how educational contexts can support students’ curiosity. The CiC framework and coding protocol, as well as tools, such as observation sheets, are openly available and can be used to pursue many of these and other future directions.

### Conclusion

This work describes the development of an observational method to assess frequency of curiosity promotion in classroom instruction. The logical next step is to use it to assess how often students experience curiosity-promoting instruction and the ways in which these experiences foster or suppress motivation, learning, engagement, and achievement. Prior work has suggested that student curiosity during classroom learning is low and decreases with grade level (Engel, 2011), and the CiC Framework coding protocol will allow researchers to observe whether curiosity promotion is happening in classrooms and whether or not it has an impact on student curiosity and learning. We describe a feasibility test of the observational protocol using video-recorded classroom instruction from a nationally representative database, showing that it is possible to measure these instructional practices. While the levels we observed in the feasibility test were low, further research is needed to explore other contexts, as well as whether this varies by subject, teacher, grade level, and many other factors. The protocol described provides a methodological tool to advance this important and needed research.

## Data Availability Statement

The raw data supporting the conclusions of this article will be made available by the authors, without undue reservation.

## Ethics Statement

The studies involving human participants were reviewed and approved by University of Virginia Institutional Review Board - Social and Behavioral Sciences. The patients/participants provided their written informed consent to participate in this study.

## Author Contributions

JJ wrote the grant proposals with the initial plan and idea for this work. JJ, SZ, and VV developed the framework and led the observational protocol work described here. JJ and SZ wrote the manuscript with important contributions from NE and VV. All authors contributed to the article and approved the submitted version.

## Funding

The research described here was supported by grants from the Spencer Foundation and the Center for Curriculum Redesign to JJ. This article was also made possible through grants from the Templeton Foundation and the Jacobs Foundation supporting this team’s broader program of research on curiosity promotion. The opinions expressed in this publication are those of the authors and do not necessarily reflect the views of the funding agencies.

## Conflict of Interest

The authors declare that the research was conducted in the absence of any commercial or financial relationships that could be construed as a potential conflict of interest.

## Publisher’s Note

All claims expressed in this article are solely those of the authors and do not necessarily represent those of their affiliated organizations, or those of the publisher, the editors and the reviewers. Any product that may be evaluated in this article, or claim that may be made by its manufacturer, is not guaranteed or endorsed by the publisher.

## Footnotes

^1^https://nyu.databrary.org/volume/1377.
